# Experiments and simulation of block motion in underwater bench blasting

**DOI:** 10.1038/s41598-023-31656-y

**Published:** 2023-03-22

**Authors:** Liang Wu, Zhijian Liang, Ming Chen

**Affiliations:** 1 Engineering Technology Research Center in Intelligent Blasting of Hubei Province, College of Science, Wuhan University of Science and Technology, Wuhan, 430065 China; 2 State Key Laboratory of Water Resources and Hydropower Engineering Science, Wuhan University, Wuhan, 430072 China

**Keywords:** Civil engineering, Geology

## Abstract

The blasting mechanism underlying drilling and blasting of underwater rocks, as an important component of the engineering blasting technology, has not been systematically studied. Laboratory model experiments are expensive and take a long time, while field tests fail to obtain timeous breakage and accumulation effects of underwater blasting, and may even be impossible. Considering this, a model experiment of underwater concrete bench blasting was designed, and the motion of blasted blocks was observed and evaluated with a high-speed camera. Then, numerical simulation was conducted based on Fluent and an engineering discrete element method coupling program complied using the application programming interface. Results show that the blocks form a bulge in the underwater blasting experiment under action of blast waves and expansion in the first period of bubble pulsation. Then, some blocks shrink in the first period of bubble pulsation. As the charge increases, the blast load exerts larger disturbance on the block group, resulting in significant motion of blasted blocks along the vertical direction. At the same time, the horizontal displacement of blasted blocks in the throwing direction increases.

Drilling and blasting of underwater rocks, as an important component of the engineering blasting technology, have been widely used to enhance the navigation capacity of river waterways, expand ports and harbors, increase waterway mileage, as well as in water intake projects and rescue and relief operations. Underwater rock blasting and ground rock blasting mainly differ in the media that are in contact with the rock surface, that is, water and air, respectively. Firstly, the acoustic impedance of water is much greater than that of air. Secondly, influences of pressure at different water depths on the yield strength of rock media need to be considered if the water is relatively deep, which is mainly shown as improvement of the resistance of rocks to tensile failure; however, these influences are negligible. In addition, broken blocks in underwater and ground blasting face extremely different resistances during throwing due to a large difference between water and air in terms of the coefficient of viscosity. Therefore, the throw distance of broken blocks blasted in air and water differs by several to tens of times.

Previous research shows that water is always present in blast holes in underwater blasting work, which avoids the direct action of detonation waves on the hole wall in rocks. This reduces the initial impact pressure peak acting on the hole wall in rocks, so crushed compression rings generally do not appear on the inner wall of blast holes. Before the blasting-induced stress waves propagate to the rock-water interface, the rock breakage mechanism is same as that in ground blasting; once the stress waves propagate to the rock-water interface, they will undergo reflection and transmission on the interface. Under these conditions, not only are compressional waves transmitted to the water, but tensile waves are reflected, appearing in the solid media, which is different from the mechanism in ground rock blasting whereby almost all incident waves are reflected to form tensile waves. Meanwhile, due to the water pressure, it is equivalent to addition of a prestress to the free surface of rocks in contact with water, which offsets the rock breakage effect of some reflected tensile stress waves. Under the confinement of water, part of the energy released after explosion needs to be allocated to overcome the water pressure. In addition, the deeper the water, the greater the confining pressure on the rock, so the extent of rock breakage is much lower than that in ground blasting under the same conditions. Therefore, to reach the same extent of rock breakage, a larger unit energy is needed and the deeper the water, the greater the energy consumption. Liu et al*.*^1^ experimentally compared the ground and underwater blasting effects using concrete samples. They found that the unit consumption of underwater blasting at a water depth of 25 m needs to increase by two to four times to reach the effect same as ground blasting; because the surface deformation of blasted rocks needs to overcome the hydrostatic pressure, water resistance also affects the throwing of broken rocks. Zhao^2^ believed that the factor that is most affected by water in underwater blasting is the throw distance. If the water depth exceeds 6 m, rocks blasted underwater will no longer fly beyond the water surface, and the throw distance of rocks in ground blasting is several and even tens of times that in underwater blasting. Theoretical analysis shows that the smaller the block, the greater the water resistance and the more impedance to throwing. The increase in the throwing velocity does not have exert a significant influence on the horizontal throw distance, while an increase in lumpiness exerts remarkable influences in increasing the throw distance.

Due to the complex influencing factors, underwater blasting can only be estimated using relevant empirical formulae and experimental data in many engineering operations, which do not model the cumulative breakage and accumulation effect. In addition, the laboratory model experiments are expensive and time-consuming, while field tests fail to obtain timeous breakage and accumulation data pertaining to underwater blasting, and may even be impossible. Therefore, more researchers are attempting to numerical methods to solve engineering problems with the development and application of improved software.

In 1980s, the discontinuous deformation analysis (DDA) method was firstly proposed^3,4^. This method can simulate blasting and throwing process of rock mass with large deformation and large displacement^5–7^. Another method is smoothed particle hydrodynamics (SPH), and it has been used in underwater blasting widely^8–13^. The SPH belongs to meshless method, and it can be coupled with other methods (the finite element method and the boundary element method) to simulate the explosion wave and bubble movement in underwater blasting. The discrete element method (DEM) is also employed to analyze the dynamic fragmentation, throwing, and the accumulated shape of rock mass under blast load^14–17^.

Nowadays, the coupling method of CFD-EDM has been adopted to calculate problems of fluid-solid coupling in engineering, including circulating fluidized beds^18^, particles motion of aerodynamic^19,20^, deep-sea mining^21^ and filter cleaning devices^22^. Based on Fluent-EDEM, Xu et al*.*^23^ have simulated the pipeline plugging process and He et al*.*^24^ have introduced the dynamic mesh. Fu et al*.*^25^ have employed the CFD-DEM method to analyze seepage collapse disaster caused by blasting in tunnel excavation. Yan et al*.*^26^ have simulated the underwater casting process of concrete with the CFD-DEM method.

In summary, the block motion involves the multi-phase coupling process of fluid-solid in underwater blasting. At present, there are still some limitations about the application of DDA and SPH to underwater blasting^27,28^. Therefore, based on application programming interface, the Fluent-EDEM coupling method will be developed further, which is a feasible scheme to study the problem of underwater blasting block movement. It is expected to promote the generalization and application of underwater blasting technology from the research findings.

## Underwater bench blasting experiments

### Experimental principle

The action of explosive waves and the quasi-static expansion of high-pressure gas are two stages of the blasting breakage process of rocks. The first stage is the rock fragmentation, in which cracks occur and penetrate the whole rock mass under action of the shock waves, stress waves, and detonation gas. The second stage is the bulges and accelerated throwing of rock mass under the quasi-static expansion of gas. In the second stage, the rocks have been fractured, forming a loose body; Therefore, it is supposed that the underwater rock mass has been fractured by blasting impact, focusing on the study of block motion under the coupled effect of detonation gas and water flow^29^.

### Preparation of the experimental model

**Figure 1:**
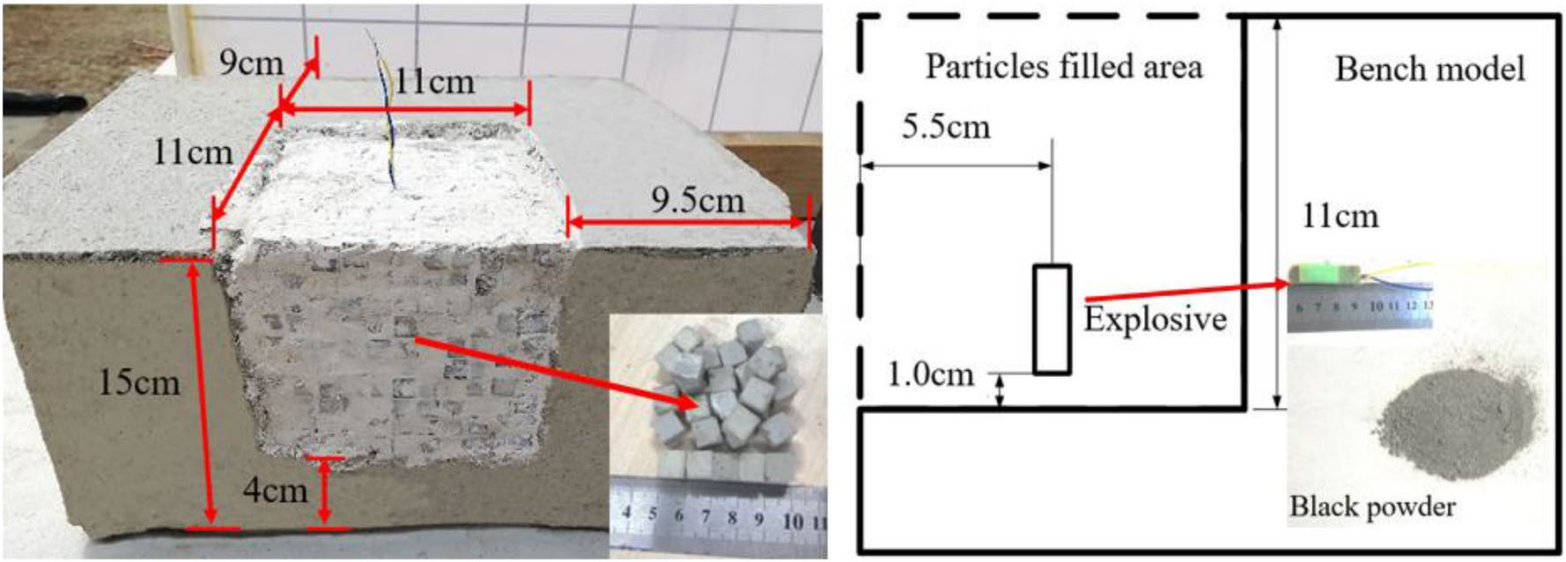
Specimen of concrete bench test and preparation of explosives.

A bench model and block particles were prefabricated according to a (C30) cement-sand-water ratio of 2:1:0.65. The prepared bench model and block particles are shown in Fig. 1. Mechanical parameters of concrete are shown in Table 1. The bench model was a cuboid measuring 300 mm × 200 mm × 150 mm, in which a space with the side length of 110 mm to be filled with block particles. The use of prefabricated blocks particles can better match the movement and accumulation of actual fragmentation. Since the size of the experimental model in this paper is limited by the size of the water tank container, the fragmentation size was calculated to be within the range of 6 ~ 11 mm according to the reference^17^. Considering that underwater blasting effect affected by water body coverage, the block size is 10 mm in this paper. Block particles of cement mortar were neatly filled in the reserved space. Steel plates with a thickness of 3 mm were used to constrain the right, left, and back faces of the bench sample.

**Table 1:** Mechanical parameters of concrete^29^.

Mass/kg	Young’s modulus /Pa	Density/kg/m^3^	Poisson's ratio	Compressive strength/Pa
0.41	2.12 × 10^10^	2.10 × 10^3^	0.25	3.23 × 10^7^

In underwater blasting project of the channel to eliminate submerged reef, the dosage of charge is used to optimize the shape of the blasting pile to improve the removal efficiency of fragmentation. To compare the motion and accumulation of block particles with different dosages of explosive, cylindrical charges of three different masses (0.8 g, 0.6 g, and 0.4 g) were prepared. Considering experimental safety, black powder was adopted as the explosive.

A concrete block was used to stem the blast hole for a length of 70 mm, so the minimum overburden of the underwater bench blasting model is 55 mm (Fig. 1). To avoid collapse of blocks filled in the space in the transportation process, a thin layer of plaster was applied to the outer surface of the blocks on the front and upper surfaces of the bench model as long as the block group does not collapse under its own weight.

**Figure 2:**
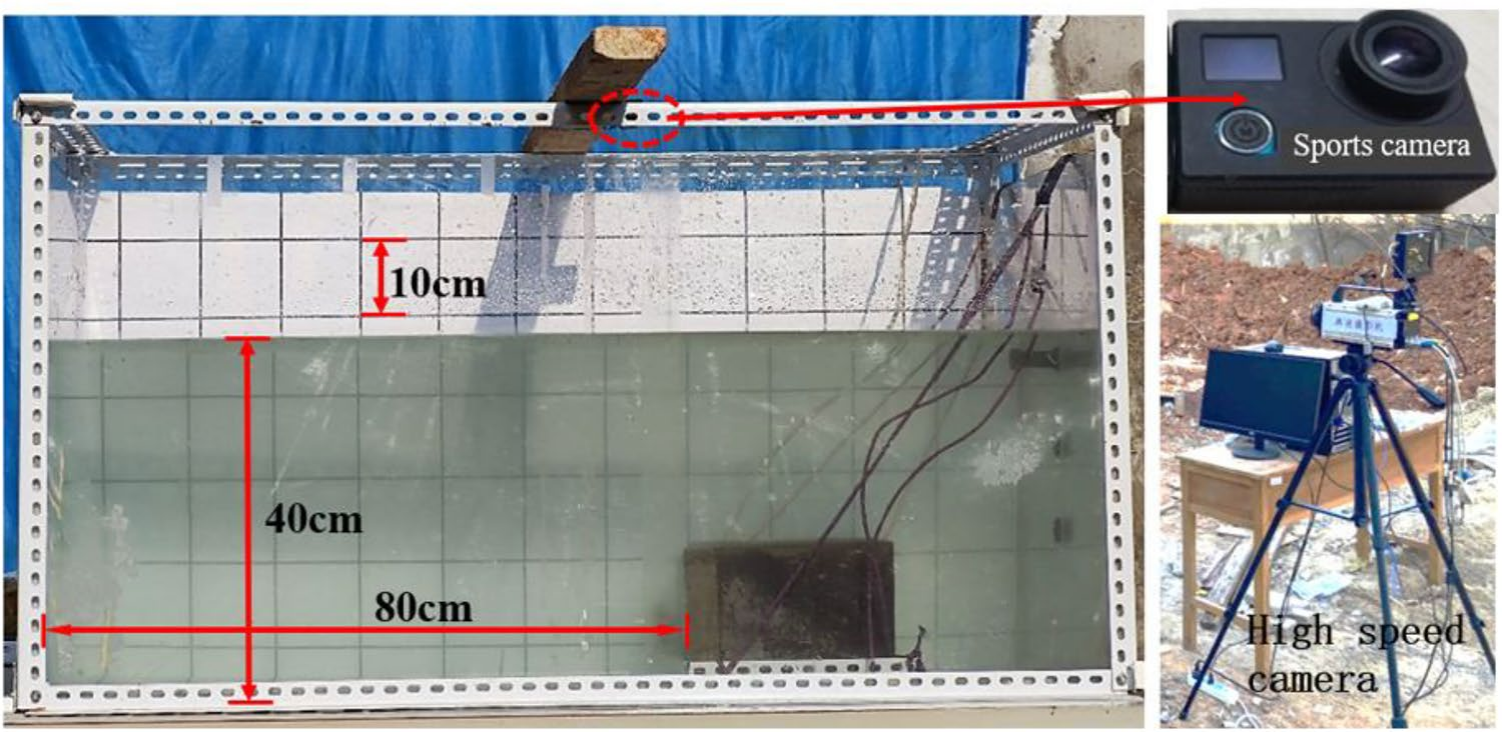
Experimental device and test system.

To observe the motion of these blocks underwater, a transparent water tank measuring 1.2 m × 0.6 m × 0.6 m was used. A GX-8 high-speed camera and a sports camera were used to record the motion process. The experimental setup and testing system are displayed in Fig. 2. In addition, a fabric background with horizontal and vertical lines (100-mm gradations) was attached on the inner face of the water tank. The water level in the water tank is fixed at 400 mm and the bench sample was placed on the central axis of the water tank. The sports camera is placed on the top of the water tank, and the high-speed camera is placed on the front of the water tank. When the concrete model is put into the water, turn on the cameras, and then trigger the explosive. The cameras will record the movement process of block particles in the water, and then the data will be saved in the computer.

## Experimental results and discussion

### Block motion process

**Figure 3:**
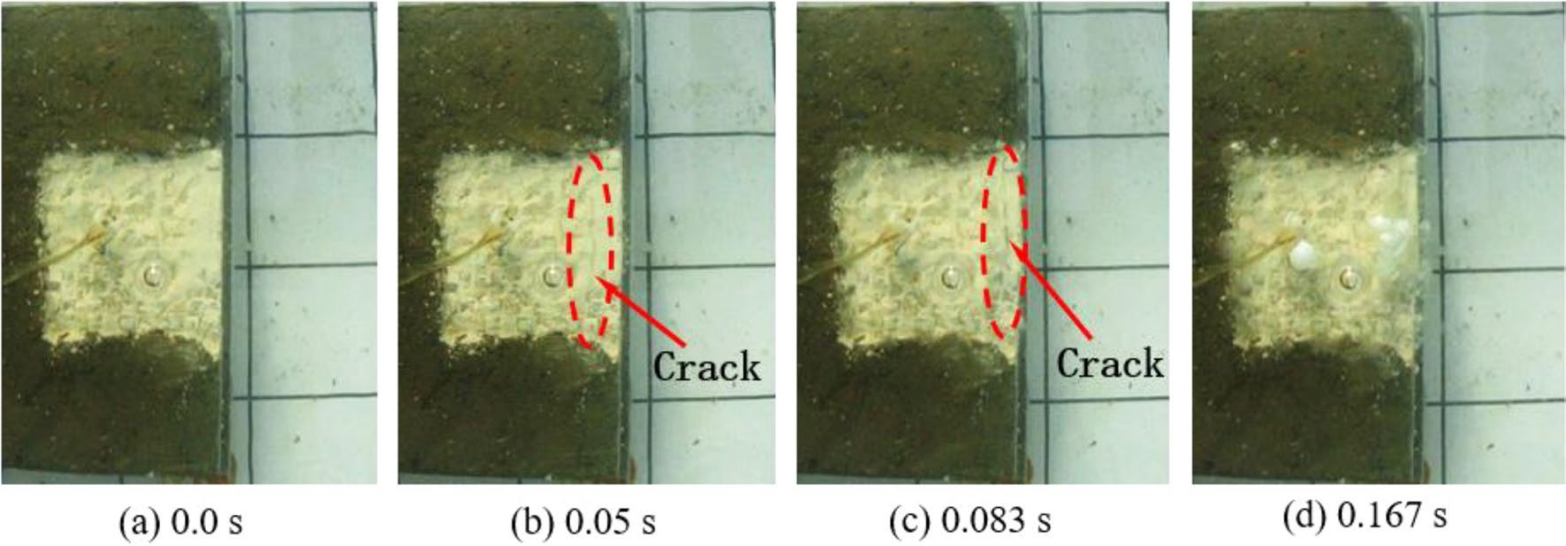
Underwater motion of blocks under a charge of 0.4 g.

**Figure 4:**
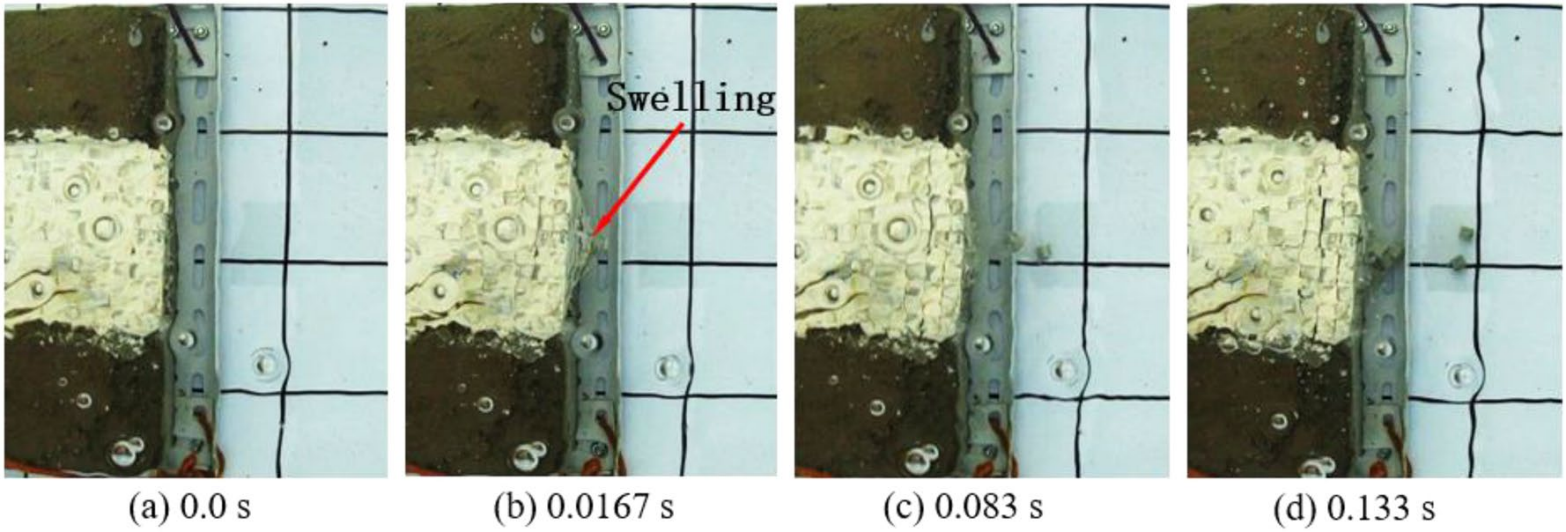
Underwater motion of blocks under a charge of 0.6 g.

**Figure 5:**
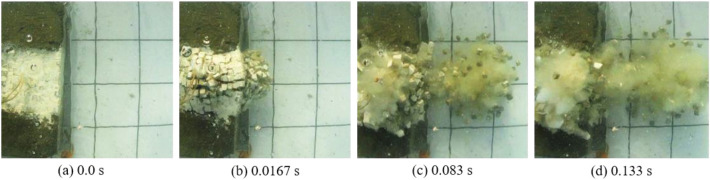
Underwater motion of blocks under a charge of 0.8 g.

The underwater motion processes of blocks under three different charges from the top view are separately shown in Figs. 3, 4 and 5. The top-view video was recorded using the sports camera. Figure 3 shows the case with the charge of 0.4 g. After detonation of the cylindrical charge, a crack begins to appear on the plaster surface on the upper surface of the bench model at 0.05 s; the second crack occurs between the outermost layer and the second layer of blocks at 0.083 s. Then, the detonation products are ejected from the cracks. Figure 4 displays the case with the charge of 0.6 g. A bulge appears on the front surface of the bench model at 0.0167 s after detonation, followed by ejection of blocks as well as failure and collapse of the block group. With the further increase in the charge to 0.8 g, the bulging of the block group increases and a much larger number of blocks is thrown out to a much greater distance (Fig. 5). It can be seen from Figs. 4 and 5 that, after the bulging of the outermost layer of blocks, the layer of blocks shrinks back then, which is more pronounced an effect when using a charge of 0.6 g (Fig. 4b,c). This is because not only blast waves affect the blocks in underwater blasting, influences of the bubble pulsation process on the blocks also need to be considered. A complete pulsation period of bubbles is composed of two stages, that is, expansion and shrinkage^30^. Therefore, the block group undergoes the following motion in the experiment: it first forms a bulge under the underwater blast waves and the expansion of the first period of bubble pulsation, and then some block particles shrink back under the shrinkage of the first period of bubble pulsation.

**Figure 6:**
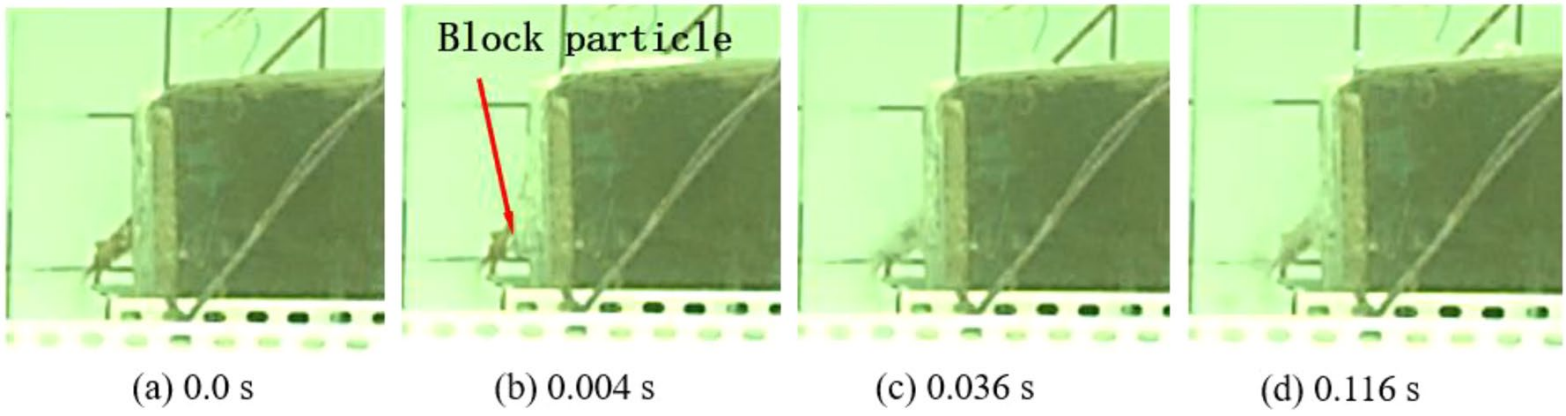
The motion of blocks under a charge of 0.6 g.

**Figure 7:**
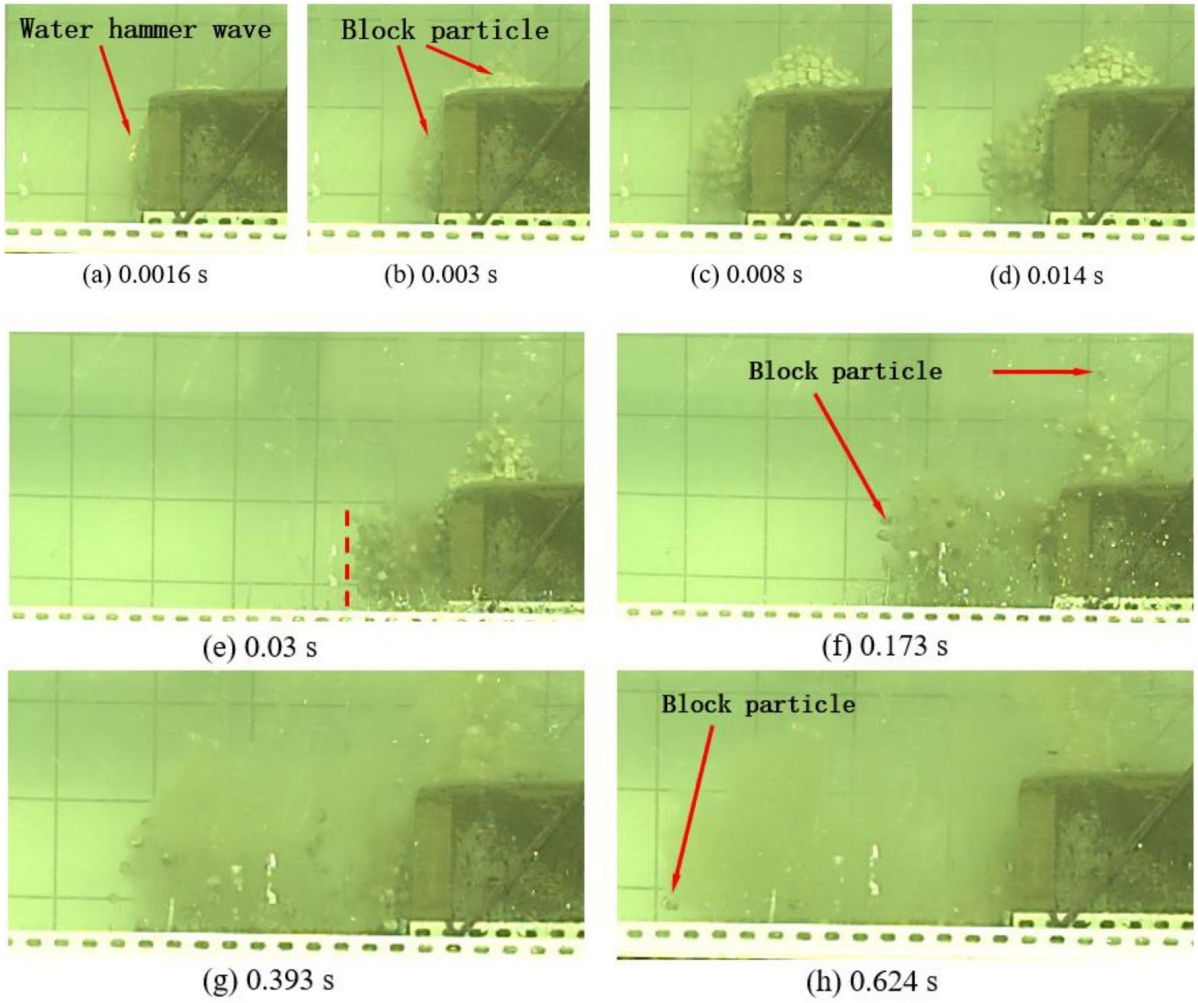
The motion of blocks under a charge of 0.8 g.

The experimental process recorded using the GX-8 high-speed camera is shown in Figs. 6 and 7. Figure 6 shows the case when the charge is 0.6 g, in which 1080 images are taken in a time period of 216 ms; because the cylindrical charge of 0.6 g is embedded in the lower part of the blocks, the bulge appears in the lower part of the bench after detonation of the cylindrical charge, while no obvious motion is found among blocks on the upper surface of the bench model. Only a few blocks are thrown out under the underwater blast waves and the bubble pulsation, and the blocks move at a low speed in the horizontal direction and they move to the farthest distance of 100 mm at 120 ms. Figure 7 illustrates the case in which the charge is 0.8 g. A total of 3634 images are taken in the process, which takes 726.8 ms. The water hammer waves can be observed on the surface of the bench at 1.6 ms; at 3.0 ms, the bulging of the block group on the surface of the bench model becomes greater than that when using a charge of 0.6 g; in the left-ward horizontal motion of blocks from the front surface of the bench model, blocks in the center move fastest and the bulge on the front surface is convex in the time period from 3 to 30 ms; the block group on the front surface of the bench is aligned at 30 ms. Before 14 ms, the block group on the upper surface of the bench further bulges and moves upward under the pressure imposed by the expanding gas; at 14 ms, some blocks on the upper surface of the bench move downward under the shrinkage of bubble pulsation and their self-weight; at 30 ms, a main passage allowing the escape of gas is formed and most blocks on the upper surface of the bench move downward, except for a few blocks, which still move upward under the action of the escaped gas; at 173 ms, blocks on the upper surface of the bench move to the greatest distance of 160 mm in the vertical direction. At 393 ms, the whole block group on the upper surface has fallen and some blocks near the lower part of the front surface also have touched the ground, stopping their motion; blocks in the middle and upper parts continue to move left-ward until touching the ground at 626.8 ms, when all blocks stop.

### Block accumulation

**Figure 8:**
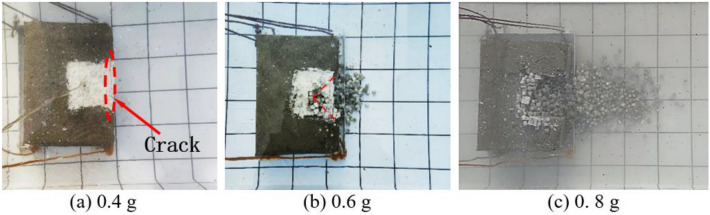
Accumulation of block particles in underwater bench blasting under different charges.

Figure 8 shows the final accumulation of blasted blocks under different charge conditions. The results indicate that most blocks are stacked ahead of the bench and a few blocks move to a greater distance mainly along the main escape direction of gas, accompanied by the Magnus effect. In addition, the horizontal motion distance is positively correlated with the charge. Figure 8a illustrates the case with a charge of 0.4 g, in which no blocks are stacked ahead of the bench model while cracks are observed on the plaster surface of the upper surface of the bench model. This indicates that the detonation of 0.4 g of explosives indeed disturbs the block particles, which, however, fails to overcome the friction between particles and between particles and inner walls of the bench as well as the hydrostatic pressure. Figure 8b shows the case with a charge of 0.6 g, in which the plaster on the upper surface of the bench model contains a blasting crater: because the cylindrical charge is embedded near the bottom of the filled area, the block layer on the upper surface of the bench does not move far; most blocks are thrown to a horizontal distance of up to 100 mm, with the furthest thrown to 150 mm. The case with a charge of 0.8 g is illustrated in Fig. 8c, in which the blocks are thrown to the furthest horizontal distance of 380 mm and most blocks to within 300 mm.

## Numerical simulation of underwater bench blasting

### CFD-DEM coupling principle and process

The exchange of interaction forces (buoyancy, lift, and drag forces) between particles and fluid realizes the coupling mechanism. According to Li^31^ and Zhao^32^, the mathematical expression of drag force is described as a function of the drag coefficient. The drag force is calculated using Eq. (1).

1 $$F_{d} = \frac{{\pi d^{2} }}{8}C_{d} \rho_{f} \left( {\user2{\mathop{v}\limits^{\rightharpoonup} } - \user2{\mathop{u}\limits^{\rightharpoonup} }} \right)\left| {\user2{\mathop{v}\limits^{\rightharpoonup} } - \user2{\mathop{u}\limits^{\rightharpoonup} }} \right|$$

2 $${C}_{d}={\left(0.63+\frac{4.8}{\sqrt{{Re}_{p}}}\right)}^{2}$$

3 $$Re_{p} = \frac{{d\rho_{f} \left| {\user2{\mathop{v}\limits^{\rightharpoonup} } - \user2{\mathop{u}\limits^{\rightharpoonup} }} \right|}}{{\mu_{f} }}$$

where *F*_*d*_ is the drag force, *C*_*d*_ is drag coefficient, and *Re*_*p*_ is Reynolds coefficient of particles. These parameters $\user2{\mathop{v}\limits^{\rightharpoonup} }$, $\user2{\mathop{u}\limits^{\rightharpoonup} }$, *d*, *ρ*_*f*_, and *μ*_*f*_, represent fluid velocity, particle velocity, particle diameter, fluid density, and dynamic viscosity of fluid respectively.

The expression of the buoyancy force is^33^:

4 $${F}_{b}=\frac{1}{6}\pi {\rho }_{f}{d}^{3}g$$

where *F*_*b*_ is the buoyancy force and $g$ is the gravity acceleration.

The expression of the lift force is:

5 $$F_{l} = \frac{1}{8}\pi \rho_{f} d^{2} \left( {\frac{1}{2}\nabla \user2{\mathop{v}\limits^{\rightharpoonup} } - \user2{\mathop{\omega }\limits^{\rightharpoonup} }} \right)\left( {\user2{\mathop{v}\limits^{\rightharpoonup} } - \user2{\mathop{u}\limits^{\rightharpoonup} }} \right)$$

where ${F}_{l}$ is the lift force and $\user2{\mathop{\omega }\limits^{\rightharpoonup} }$ is the angular velocity of particles.

By compiling the application programming interface (API), the energy and momentum are transferred between Fluent and DEM. The API contains the coupling scheme with fluid-particle interaction forces. At the beginning, EDEM program is run first to set up the initial state of particles. Subsequently, by the coupling interface program, Fluent obtains the particle status information transmitted by EDEM, then updates the grid state of fluid and attains the interactional forces. Thereafter, the interactional forces (the drag, lift, and buoyancy forces) are transferred to the EDEM by the API to update the state of particles. Finally, the next cycle starts.

### Calculation model

**Figure 9:**
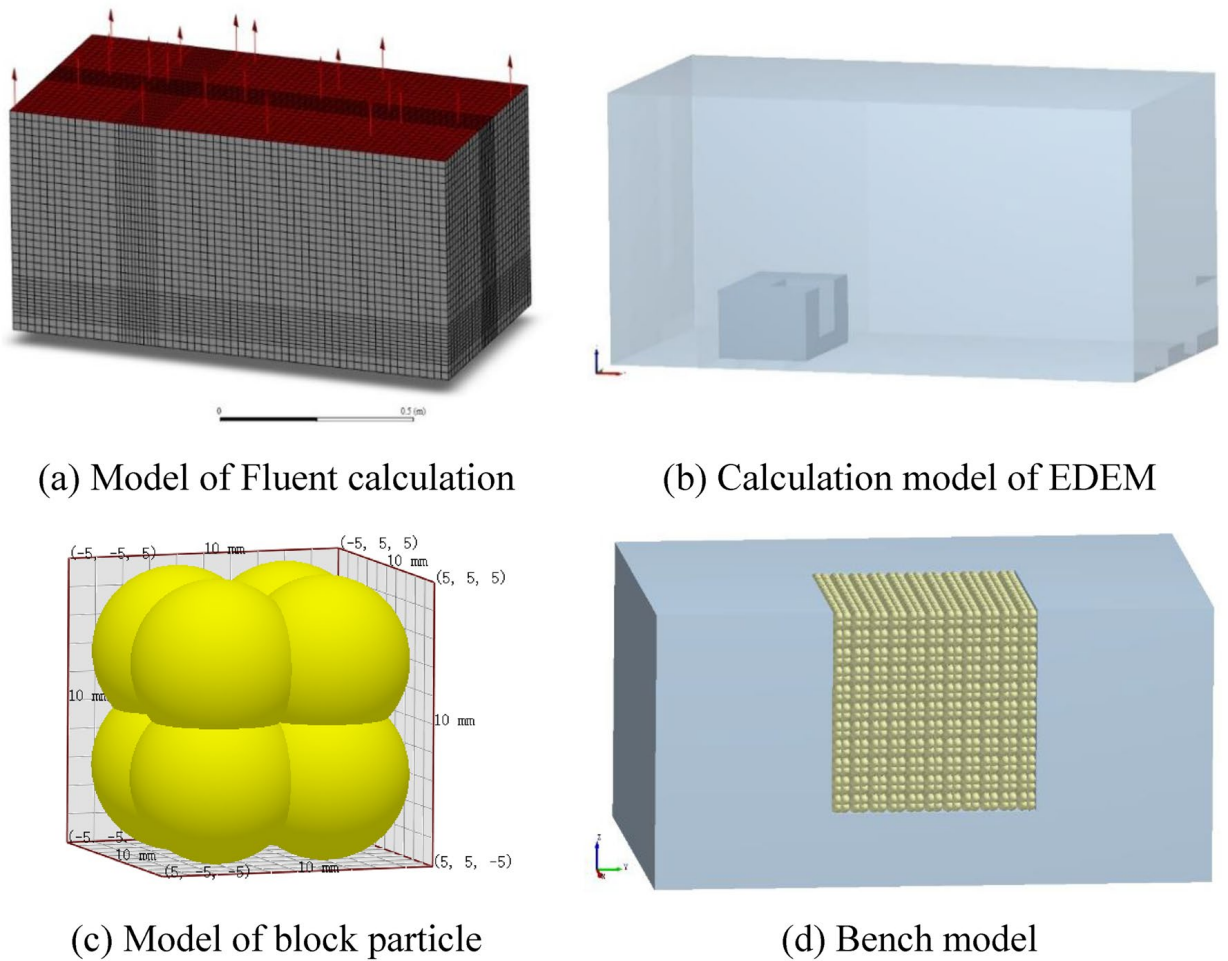
Calculation models in the simulation.

The numerical model established in reference to the experimental set-up is illustrated in Fig. 9. Figure 9a,b separately show the underwater bench blasting Fluent model and the EDEM model, and the calculation model shares the same dimensions as the water tank in the experiments. The multi-phase flow is opened in the Fluent and materials of air and liquid water are added in the material library of Fluent. The high-pressure energy source acts on the surrounding solid particles through the fluid in the blast hole, thus realizing the simulation of the explosion load acting on the blast hole wall. A pressure outlet is established on the upper surface of the model with a gauge pressure set to atmospheric pressure; other boundaries are set as non-slip surfaces. Except for the high-pressure area for simulating the cylindrical charge that is refined with 10-mm grids, the side length of grids in the fluid region is 20 mm. Figure 9c depicts the EDEM particle model established in the simulation, with the overall dimensions match those of the concrete block particles in the experiments (*i.e*. particles with a side length of 10 mm). The block particles model is constituted from eight spherical particles. Material parameters of the particles and bench walls are listed in Table 1, while materials for the wall of the water tank are listed in Table 2. To conform to the filling condition of block particles in the filling area of the bench model, the particle factory program is compiled using the API function of EDEM. This allows particles to be neatly accumulated in the calculation model of the bench, as shown in Fig. 9d, in which a total of 1331 particles are filled. The time step of the EDEM is 1 × 10^–6^ s, while that of the Fluent model is 50 times that (5 × 10^–5^ s).

**Table 2:** Parameters of the EDEM model^29^.

Material parameters	Density /kg/m^3^	Young’s modulus /Pa	Poisson's ratio	Coefficient of recovery	Coefficient of static friction	Coefficient of rolling friction
The wall of water tank	1.20 × 10^3^	2.94 × 10^9^	0.25	0.12	0.2	0.01
The wall of bench, blocks	2.10 × 10^3^	2.12 × 10^10^	0.25	0.12	0.5	0.01

### Parameter calibration

The basic mechanical parameters required in the EDEM calculation model include the bench specimen, particle materials and the material of water tank. The friction coefficient between particles, and the particles and wall surfaces that may contact should also be set. The experiment of using discrete particles to accumulate under the action of self-weight to form a stable slope is called the experiment of repose angle. Its purpose is to calibrate the friction coefficient between particles according to the natural repose angle of the slope^34–36^.

**Figure 10:**
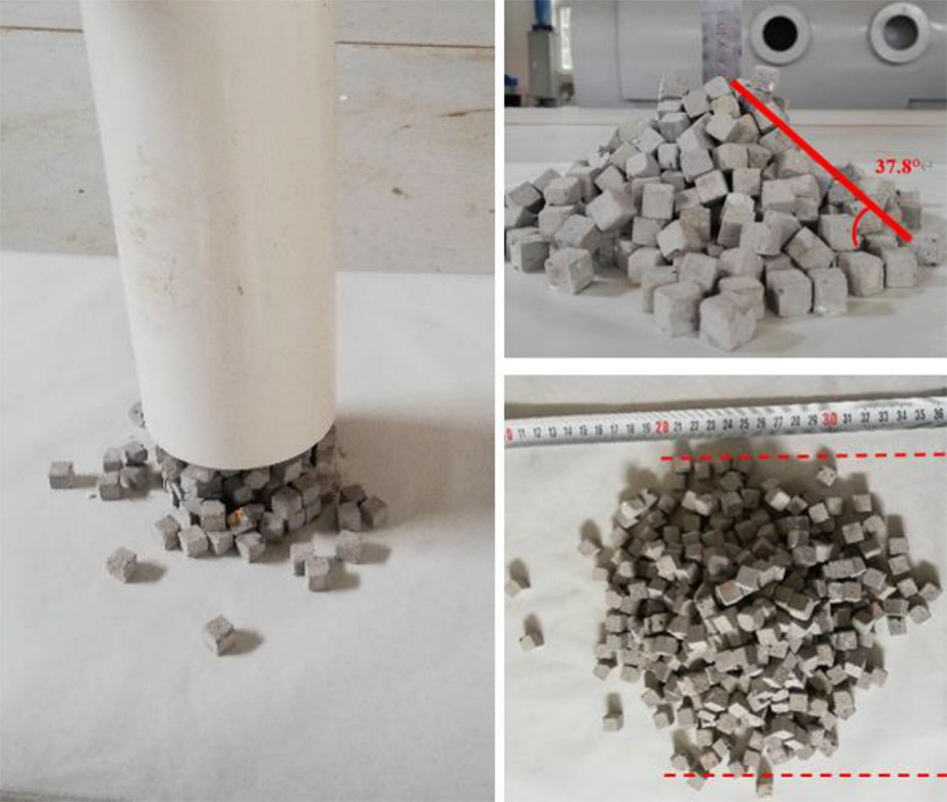
The angle of repose test.

The experiment process of the repose angle is illustrated in Fig. 10. Firstly, a hollow cylinder with an inner diameter of 80 mm is placed vertically on a horizontal plane, and then the block particles are randomly filled into the hollow cylinder to the height of 150 mm. The cylinder is lifted at a constant speed along the vertical direction, and the particles fall freely to form a pile. Finally, the repose angle of the pile is measured, with an angle of about 38°.

**Figure 11:**
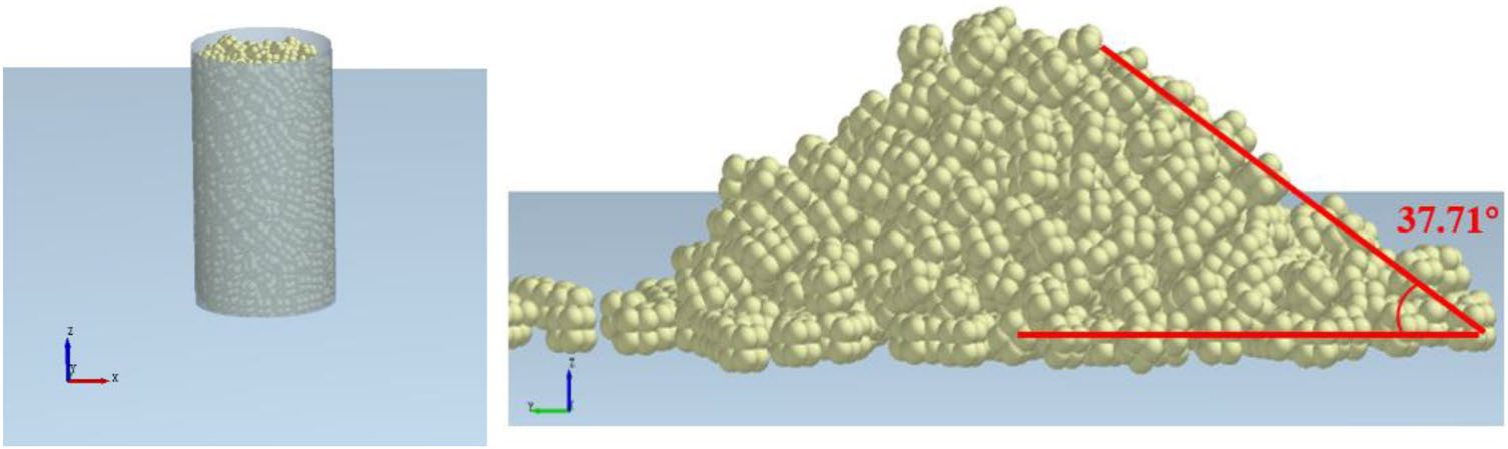
Simulated angle of repose in the EDEM.

A hollow cylinder model with a height of 150 mm and the diameter of 80 mm is established in the EDEM, and the particles (model in Fig. 9) are randomly filled into the hollow cylinder to the height of 150 mm. Then the cylinder is lifted. Finally, the repose angle of the particle group is calculated, and the friction coefficient is determined by comparing the numerical simulation with the experimental results, as is displayed in Fig. 11. The coefficients of friction in the model are summarized in Table 2.

## Simulation results

### Block motion process

**Figure 12:**
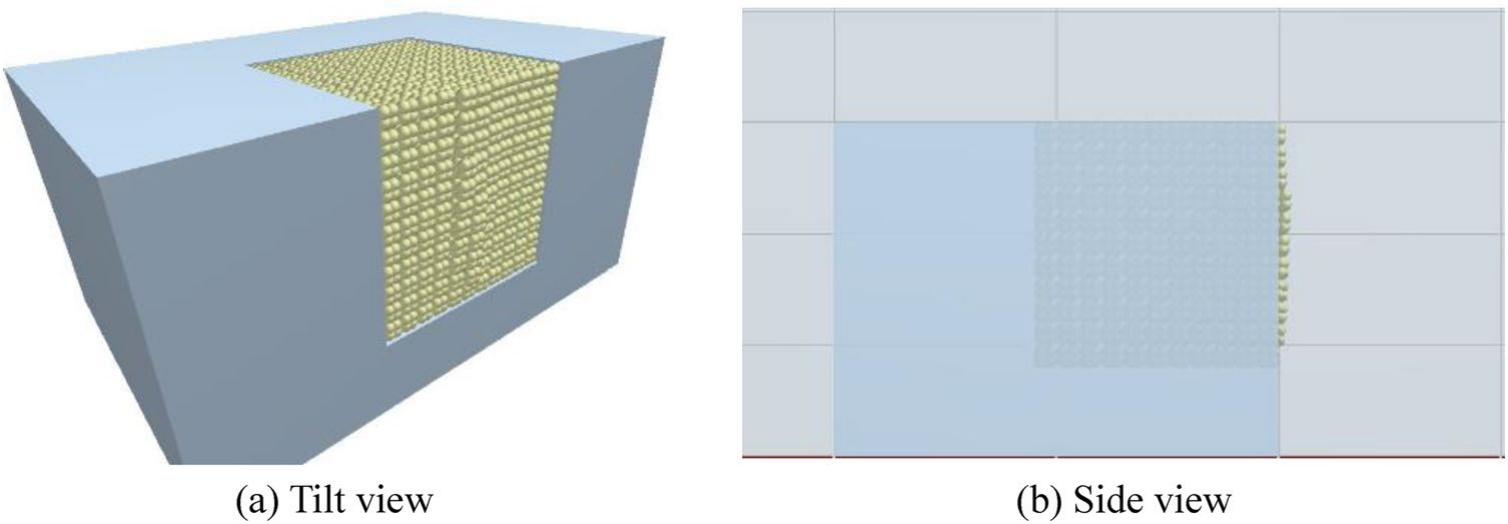
The motion of the blocks (0.4 g).

A high-pressure area is set in the same region of the calculation model according to the position of the cylindrical charge in Fig. 1. The case with the charge of 0.4 g is illustrated in Fig. 12. It can be seen from the figure that blocks in the middle of the bench tend to move outwards, while the sliding friction force between blocks and the water pressure suppress the further progress thereof. The side view of the bench under the charge of 0.4 g shows that only the outermost layer of blocks on the front surface of the bench model undergoes any outward displacement and most blocks are not displaced (Fig. 12b). The length and width of elements in Fig. 12b are 100 and 50 mm, respectively.

**Figure 13:**
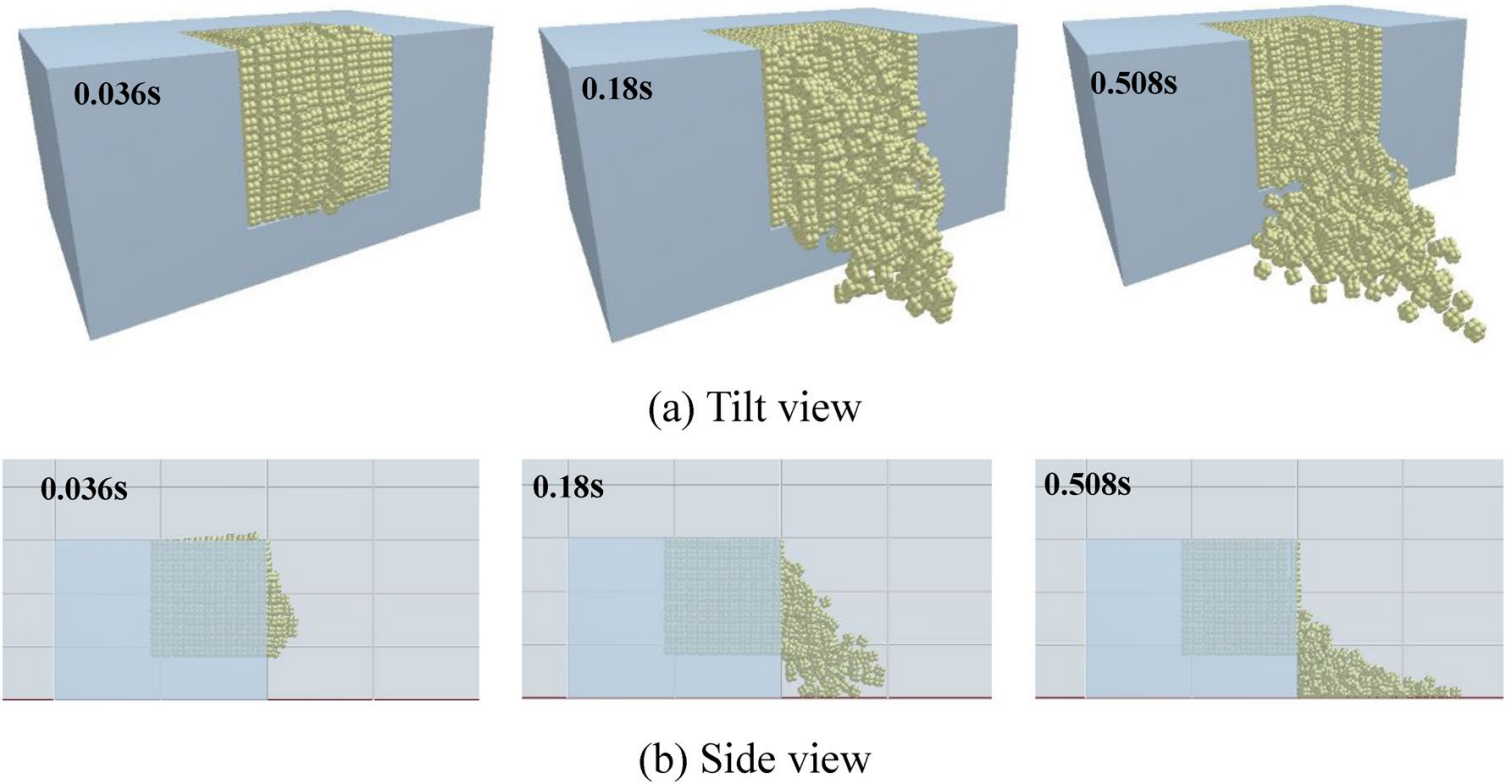
The motion of the blocks (0.6 g).

Figure 13 displays the calculation results when the charge is 0.6 g. After the simulation begins, the blocks on the upper surface of the bench model fluctuate while the whole block group is dominated by the motion of layers of block particles on the front surface. The motion of the block group on the front surface of the bench can be divided into two types: the bulging of the outermost layer of blocks on the front surface in the burden direction under explosion, which lasts for a short time and the blocks touch the ground at 180 ms; the other is the collapse and tumbling of the subsequent block group which finally forms a stack ahead of the bench, because of the formation of a new free face after ejection of the outermost layer of blocks on the front surface and the disturbance induced by energy released from the high-pressure area. The collapsed and tumbled blocks are located ahead of the high-pressure area before motion while those behind the area remain practically motionless. Figure 13b shows the motion of blocks from the side view under conditions with the charge of 0.6 g: the bulging of the blocks is mainly found in the lower part of the bench because the charge is set low in the experimental model, so the high-pressure area is also around the same location in the simulation. As a result, the blocks in the lower part on the front surface of the bench gain more energy than those in the upper part.

**Figure 14:**
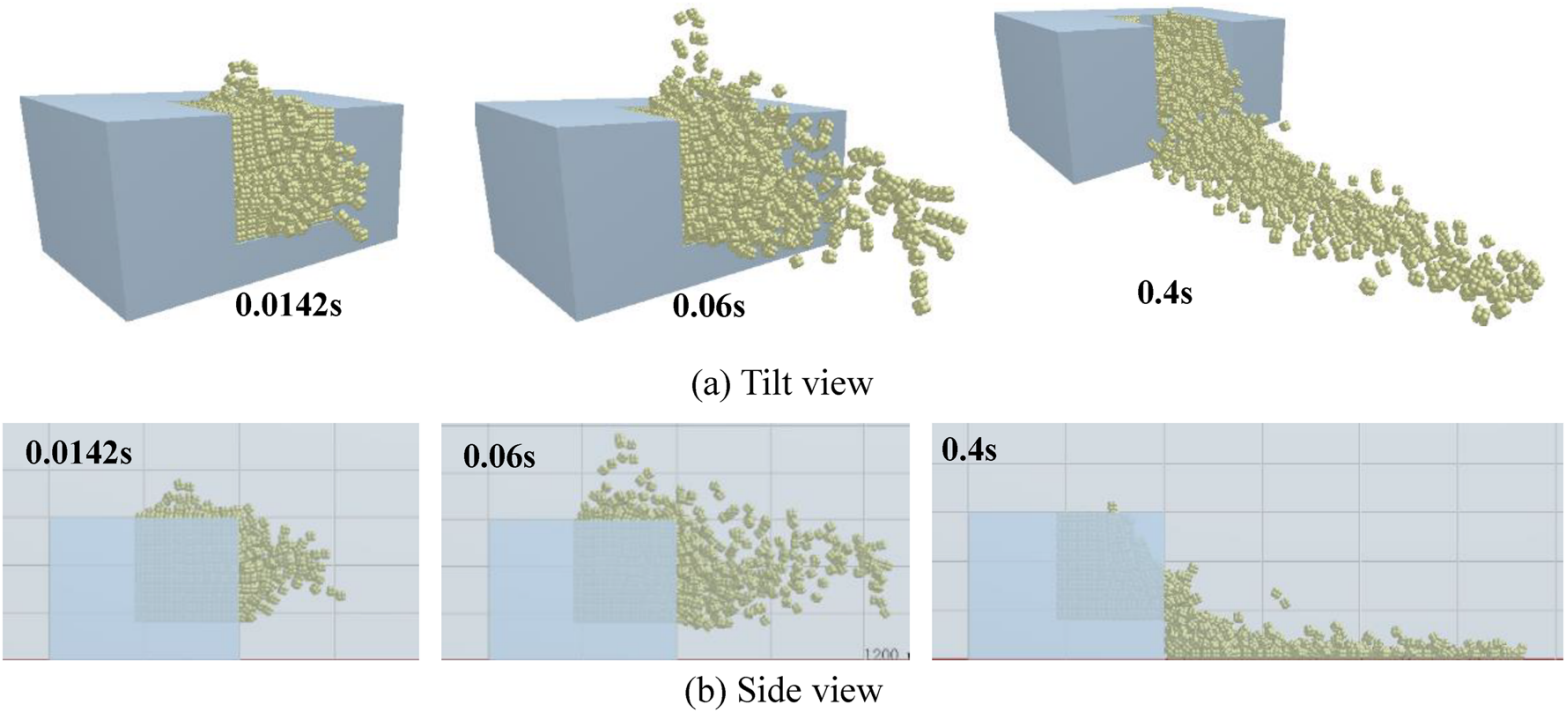
The motion of the blocks (0.8 g).

Figure 14 demonstrates the calculation results under a charge of 0.8 g, in which motion of blocks is more intense than under the other two charge conditions. Bulges are seen at 8 ms on both the upper and front surfaces of the bench. The blocks on the upper surface move to their maximum height at 172 ms and then begin to fall, while the block group on the front surface continues to move forward, in which some blocks have become grounded. The blocks on the upper surface have fallen by 392 ms, when only a few blocks which are thrown along the direction of the overburden keep moving in the water while most blocks moving in that direction have fallen. Blocks behind, and on both sides of, the blast hole move, and form a stack in the blasting crater through collapsing and tumbling. Figure 14b shows the side view of the motion of blocks under a charge of 0.8 g. With the increasing pressure in the high-pressure area, the blocks on the upper surface of the bench model also form a bulge. Compared with the calculated results under a charge of 0.6 g, the blocks in the direction of the overburden move faster under a charge of 0.8 g. The blocks have reached the scale mark at 100 mm by 14.2 ms, matching the experimental observation made using the high-speed camera.

### Range of accumulation of blocks

**Figure 15:**
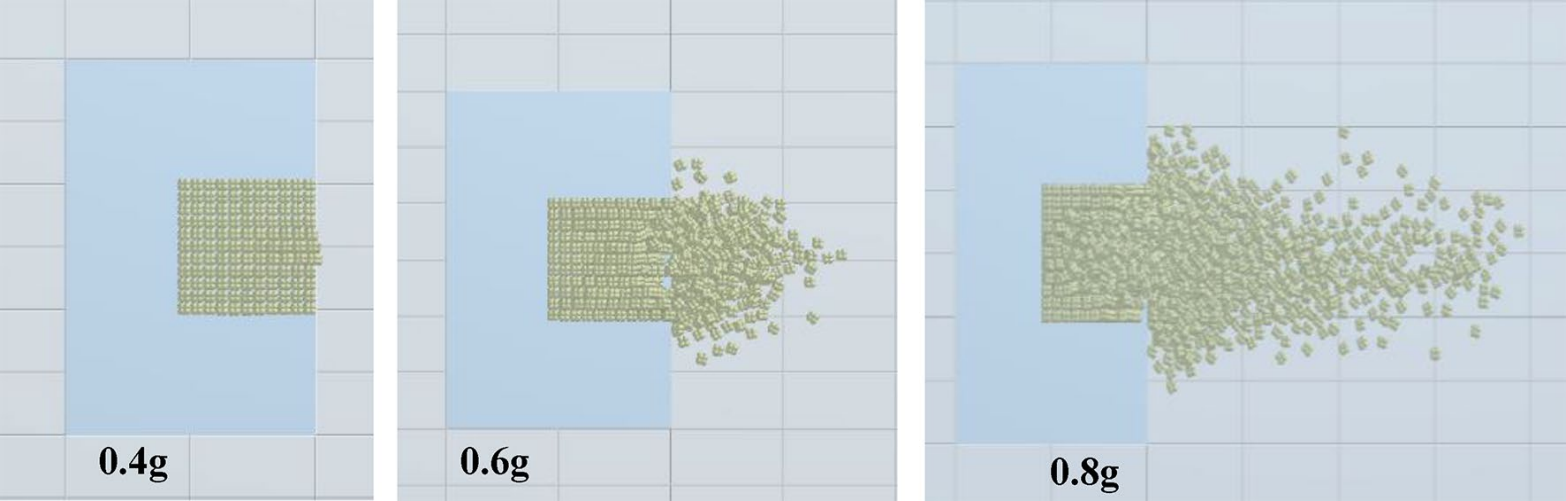
The final accumulation of block particles under different charges from the top view.

Figure 15 shows the final accumulation of block particles in the numerical simulation under different charges from the top view. Under a charge of 0.4 g, blocks fail to overcome the friction between blocks and the water pressure despite being under the load applied to the high-pressure area, while only the outermost layer of blocks on the front surface of the bench model shows outward displacement. The result is consistent with the experimental result. By combining Figs. 13 and 15, the simulation results under a charge of 0.6 g indicate that the charge mainly plays a role in loosening the block group; blocks on the front surface of the bench model differ slightly in term of distance travelled, and all blocks are found within 150 mm ahead of the front surface of the bench model; because the high-pressure area is located in the lower part of the bench, blocks are not vertically ejected from the plugged part, which is consistent with the experimental result. Under a charge of 0.8 g, the blocks move to the furthest distance of 388 mm while mainly accumulating in the range from -100 to 100 mm, basically coinciding with the accumulation of blocks seen in the experiment.

### Velocity and layered accumulation of blocks

**Figure 16:**
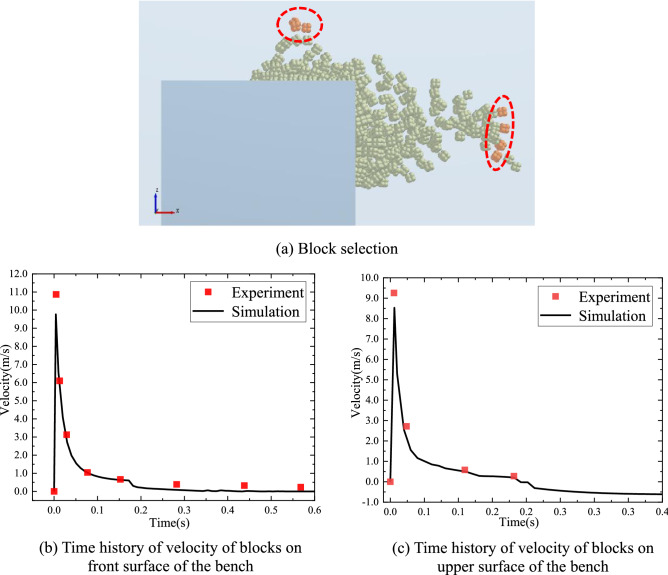
Comparison between the simulated and experimental curves (0.8 g).

To discuss the velocity of blocks, the charge condition of 0.8 g was studied. Several blocks were selected from the front and upper surfaces of the bench model, as shown in Fig. 16a. Figure 16b,c display the time histories of the velocities of blocks on the front and upper surfaces of the bench model, respectively. The numerically calculated velocity curve of blocks is the average velocity curve of blocks selected in Fig. 16a, while the experimental curve is a scatter graph obtained using the high-speed camera. The calculated horizontal velocity of blocks in the burden direction in Fig. 16b reaches the maximum of 9.77 m/s at 4 ms, while that of the experimental curve reaches the maximum of 10.87 m/s at 4.6 ms. Figure 16b shows the velocity curves of blocks moving vertically to the upper surface of the bench. The blocks reach a peak velocity of 8.53 m/s at 6 ms according to numerical calculation, while they reach the peak velocity of 9.26 m/s at 5.4 ms in the experiment. The results indicate high consistency between the numerical and experimental results.

**Figure 17:**
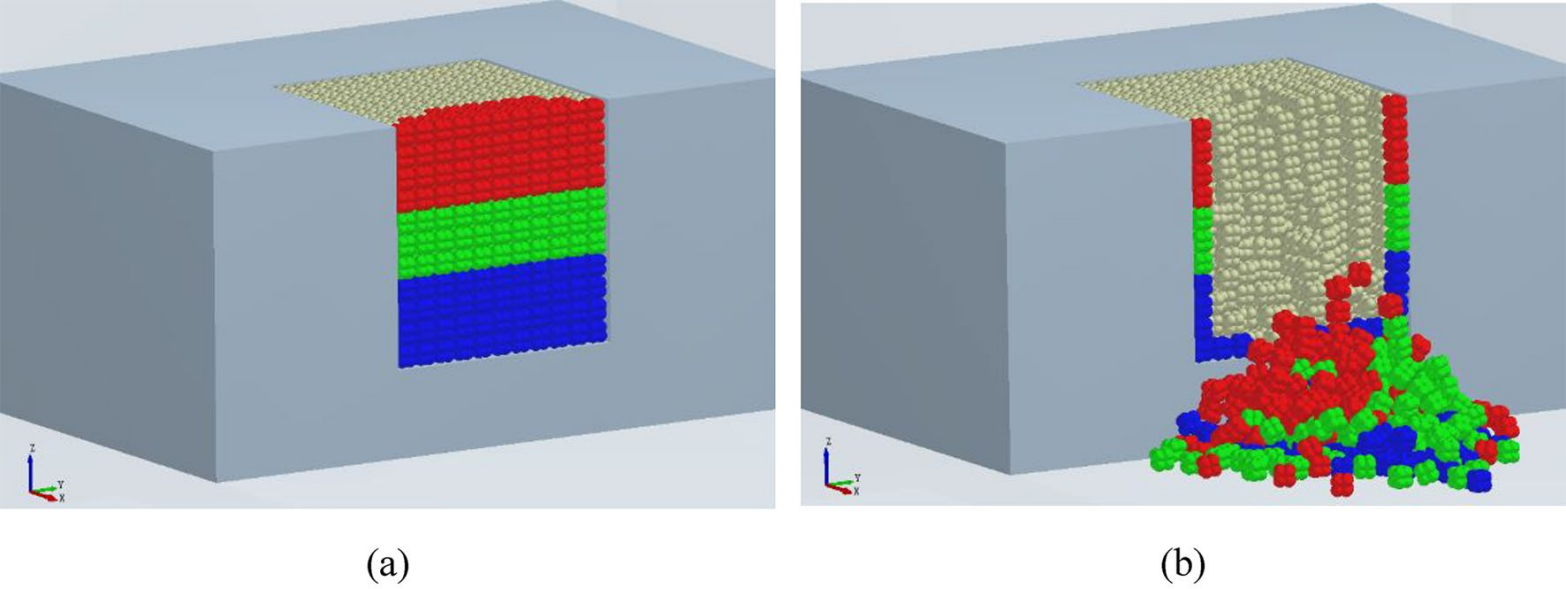
Layered accumulation of blocks (0.6 g).

**Figure 18:**
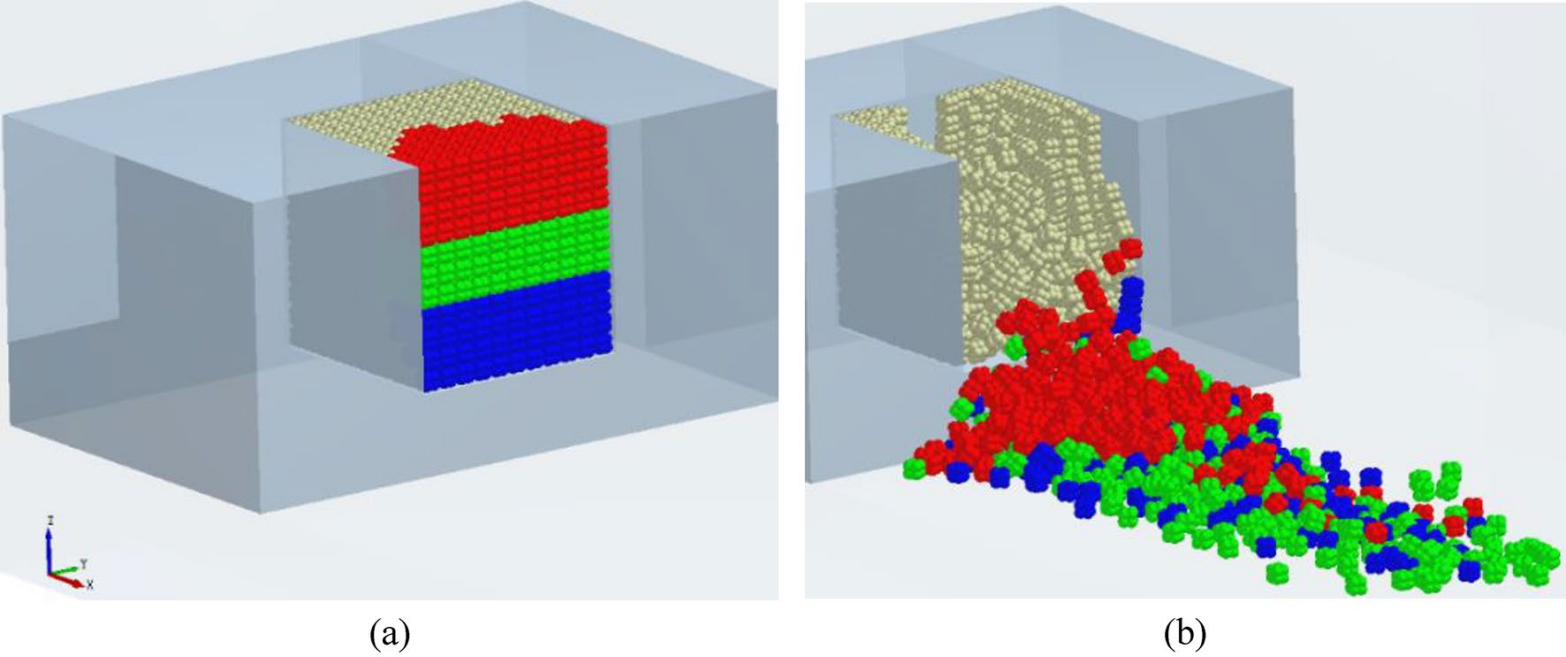
Layered accumulation of blocks (0.8 g).

To discuss the layering characteristics of block accumulation, the thrown and accumulated blocks under charges of 0.8 g and 0.6 g were colored, as shown in Figs. 17 and 18, in which blocks in the lower, middle, and upper parts are marked with blue, green, and red, respectively. As can be seen from Figs. 17a and 18a, the block particles accumulated ahead of the bench all come from the blasting crater. Results in Figs. 17b and 18b indicate that the layering of accumulated blocks allows them to maintain their original relative positions: the blue blocks are at the bottom; green ones are accumulated on the blue blocks and they are thrown the furthest due to their initial high location and high velocity; red blocks move at a relatively low horizontal velocity despite their highest location, so they are accumulated atop the others.

## Conclusions

Underwater bench blasting experiments of small explosive charges were conducted. The high-speed camera was used to observe and study the motion of blocks in underwater blasting. Moreover, the Fluent-EDEM method was used for numerical simulation, which verified the feasibility and accuracy of the method. The following conclusions are obtained:

1. The blasted blocks form a bulge in the underwater bench blasting experiment under action of the blast waves and the expansion in the first period of bubble pulsation. Then, some blocks shrink back under the shrinkage of the first period of bubble pulsation.
2. The numerical simulation results indicate that the blast load produced by a charge of 0.6 g mainly loosens the blocks, while the blocks are thrown further when using a charge of 0.8 g. This finding suggests that the blast load exerts greater disturbance on the block group with the increase in the charge, leading to greater motion of blocks in the vertical direction. Meanwhile, the horizontal displacement of blocks in the throwing direction also increases.
3. The numerical simulation results are highly consistent with the experimental results in terms of the accumulation and motion velocity curves of blocks. This indicates that the coupling method can accurately simulate underwater bench blasting.

## Data Availability

All data generated or analysed during this study are included in this published article.
